# Rainfall shocks are not necessarily a sensitive early indicator of changes in wasting prevalence

**DOI:** 10.1038/ejcn.2017.144

**Published:** 2017-09-13

**Authors:** N A Ledlie, H Alderman, J L Leroy, L You

**Affiliations:** 1 International Food Policy Research Institute (IFPRI), Washington, DC, USA

## Abstract

Evidence on the impact of weather shocks on child nutrition focuses on linear growth retardation (stunting) and thus, associates the effect of a short-term measure (weather events) on a cumulative measure (attained height). Relatively little is known on how weather shocks predict increases in wasting in a population. This study explores whether deviation in rainfall in Ethiopia, a drought prone country, is a sensitive indicator of future increases in wasting. Around 12% of children 0–23 months were wasted, but we found no consistent association between the rainfall shock variables and child weight-for-height *Z*-scores. The results indicate that monitoring rainfall does not provide a practical early warning to use for scaling up financing and management of preventative measures without additional information to increase precision.

## Introduction

An estimated 52 million children worldwide suffer from acute malnutrition which dramatically increases their risk of death: compared to well-nourished children, children with a weight-for-height *Z*-score (WHZ) 2–3 s.d. below the median die at a rate three times higher than those with a WHZ of −1 or higher.^1^ Equally telling, roughly half of all the projected lives saved from scaling up 10 key nutrition specific interventions would come from improved management of acute malnutrition.^2^

It is relatively difficult to assess acute malnutrition on a population scale since it is more time sensitive than either stunting or underweight and thus harder to track in periodic household surveys.^3^ Indicators of undernutrition are elevated in times of climatic shocks,^4^ a concern both for tracking of nutritional status and for designing appropriate programs. With a few exceptions,^5^ however, the evidence on the impact of weather shocks on child nutrition focuses on linear growth retardation (stunting) and thus, uses a short term, acute weather event to explain a cumulative measure of nutrition. This reflects a misunderstanding of a biological pathway for height determination, and may lead to incorrect interpretation of the impacts of weather shocks on child nutritional status.^6^ Although there exist protocols for screening and effective treatment for (severe) acute malnutrition, relatively little is known on how weather shocks predict increases in wasting within a population. To encourage timely and informed action, this study explores whether deviation in rainfall in a drought prone country, Ethiopia, is a sensitive indicator of future increases in wasting.

## Methods

Our data came from the 2011 to 2012 and 2013 to 2014 rounds of the Ethiopia Socioeconomic Survey which were collected as a part of the Living Standards Measurement Study-Integrated Surveys on Agriculture (LSMS-ISA) project of the World Bank. The sample was drawn from a population frame that included all rural and small town areas of Ethiopia except for three zones of Afar and six zones of Somali region.^7^ In each household, the survey collected socioeconomic, demographic and nutrition-related information at both the household and individual levels. We focus on a subset of 2659 households with a child 6–59 months old. The household data were supplemented with monthly rainfall data for years 2011 and 2013 covering Ethiopia five arc-min spatial resolution, that is, squares of ~9.27 × 9.27 km at sea level.^8^ While Ethiopia has two rainy seasons, more than 90% of total grain production comes from the harvest following the long rains. Thus, we focus on that season.

The effects of rainfall shocks on child WHZ were estimated using ordinary least squares regression (using Stata 14). Child WHZ was calculated using the WHO growth standard.^9^ Rainfall shocks were defined for the long rainy season (June through September) by calculating cell-specific rainfall Z-scores, that is, the number of s.d. from the local 13-year rainfall mean. We also constructed a binary variable to identify those localities where rainfall was less than 90% of normal. We used ordinary least squares regression assuming the residuals to be independent and identically and normally distributed. All models adjusted for demographics (child sex and age, household size and parent’s education) and household wealth. An index of household wealth was constructed from measures of quality of housing (materials used for flooring, walls, and roof; ownership of a kitchen, number of rooms, type of drinking water and toilet and the availability of electricity) and asset ownership (functioning radio, television and so on), using Stata’s factor command for principal component factor analysis. Scores from the first factor, which explained 17% of the total variance, were used to create the wealth variable. Factor weights from the 2011 survey were applied to the 2013 survey so that the index was comparable across surveys. Models adjusted for household poverty by using binary indicators of the quartiles of the constructed wealth index. Standard errors were adjusted using Huber Sandwich estimators of variance.

## Results and discussion

Around 11% of the children were wasted which is defined as a serious public health problem by the WHO. Rainfall-related shocks were common, affecting ~18% of the observations (Table 1). Despite the ample variation in rainfall both spatially and temporally, we found no association between the rainfall shock variables and child WHZ either for children under 24 months (Table 2) or for children 24–59 months (Supplementary Table). Given that wasting was associated with wealth (*P*<0.05 in younger and *P*<0.1 in older children) and maternal education (*P*<0.05 in older children) suggests that the issue is not driven by insufficient power.

Although a recent systematic review indicated that 12 of 15 studies found an association of weather events and stunting,^10^ this current study provides nuance in regards to timing. While both surveys were conducted within 4 months of the harvest—perhaps a partial explanation for absence of a significant association—the point estimates of the month dummies (a proxy for time since harvest) decline as time increase, although these coefficients are not significant as determinants of child WHZ.

We make no claims on the external validity of this result and doubt that it challenges the general concern about climate change and weather shocks on child malnutrition. Adverse weather affects both farm incomes and consumer prices and thus would have longer term impact on nutrition among low income households through such price shocks. However, the results do indicate that monitoring rainfall does not provide a practical early warning to use for scaling up financing and management of preventative measures without additional information to increase precision.

## Figures and Tables

**Table 1:** Characteristics of the study sample^a^

	*Children 6–23 months*	*Children 24–59 months*
*Child characteristics*		
Age (months)	14.69±4.80	40.89±10.00
Child sex, male (%)	51.15	51.79
Weight-for-height *Z*-score	−0.18±1.70	−0.40±1.45
Wasting (%)	11.82	10.30
		
*Parental education*		
Mother, some education (%)	30.05	25.24
Father, some education (%)	52.38	46.60
		
*Household characteristics*		
Size	6.23±2.21	6.37±2.19
Access to a toilet (%)	54.76	56.83
		
*Rainfall characteristics*		
*Long rain*		
* Z*-score	−0.14± 0.56	−0.14±0.55
Decrease >10% (%)^b^	18.39	17.64
Observations	1218	2659

a Values are mean±s.d. or %.

b ‘Decrease >10%’ was calculated as the percent of children in clusters where the fall in rainfall was >10% of the mean for that cluster.

**Table 2:** Association of individual, household and rainfall characteristics with child weight-for-height *Z*-scores (children 6–23 months)^a^

*Rainfall shock*	*Z-score*	*Decrease >10%*
Mother's education	−0.03	−0.03
	(0.108)	(0.108)
Father's education	−0.02	−0.02
	(0.023)	(0.023)
Wealth index: Quartile 2	0.35**	0.35**
	(0.138)	(0.137)
Wealth index: Quartile 3	0.51***	0.50***
	(0.146)	(0.146)
Wealth index: Quartile 4	0.41***	0.41***
	(0.142)	(0.142)
Month dummy=February	0.21	0.20
	(0.471)	(0.470)
Month dummy=March	−0.19	−0.19
	(0.525)	(0.524)
Month dummy=April	−0.65	−0.63
	(0.484)	(0.485)
Rainfall shock	0.01	0.04
	(0.089)	(0.129)

a Values are regression coefficients and standard errors. Other covariates included in the model were child age and sex, household size and a year dummy (see Supplementary Table for full results). ***P<0.01, **P<0.05, *P<0.1.
