# Supplementary material for: Rainfall shocks are not necessarily a sensitive early indicator of changes in wasting prevalence
Source: Eur J Clin Nutr. 2017 Sep 13;72(1):177–8. doi: 10.1038/ejcn.2017.144 (PMC5765168; doi:10.1038/ejcn.2017.144)
Supplement: Supplementary Table [file ejcn2017144x1.docx]

Supplemental Table: Association of individual, household, and rainfall factors with child weight for height z-scores

|  | (1) | (2) | (3) | (4) |
| --- | --- | --- | --- | --- |
| *Child age* | *6-23 months* | *6-23 months* | *24-59 months* | *24-59 months* |
| *Rainfall shock* | *Z-score* | *Decrease greater than 10%* | *Z-score* | *Decrease greater than 10%* |
| *Child characteristics* |  |  |  |  |
| Age in months | 0.01 | 0.01 | -0.00 | -0.00 |
|  | (0.010) | (0.010) | (0.003) | (0.003) |
| Child sex, male (%) | -0.01 | -0.01 | -0.06 | -0.06 |
|  | (0.097) | (0.098) | (0.056) | (0.056) |
| *Parental education* |  |  |  |  |
| Mother's education | -0.03 | -0.03 | 0.17*** | 0.17*** |
|  | (0.108) | (0.108) | (0.065) | (0.065) |
| Father's education | -0.02 | -0.02 | 0.03 | 0.03 |
|  | (0.023) | (0.023) | (0.022) | (0.021) |
| *Household characteristics* |  |  |  |  |
| Wealth Index: Quartile 2 | 0.35** | 0.35** | 0.08 | 0.08 |
|  | (0.138) | (0.137) | (0.084) | (0.084) |
| Wealth Index: Quartile 3 | 0.51*** | 0.50*** | 0.15* | 0.15* |
|  | (0.146) | (0.146) | (0.082) | (0.082) |
| Wealth Index: Quartile 4 | 0.41*** | 0.41*** | 0.14* | 0.14* |
|  | (0.142) | (0.142) | (0.081) | (0.081) |
| Household size | -0.01 | -0.00 | 0.01 | 0.01 |
|  | (0.022) | (0.022) | (0.013) | (0.013) |
| *Interview month dummies* |  |  |  |  |
| Month dummy = February | 0.21 | 0.20 | -0.16 | -0.16 |
|  | (0.471) | (0.470) | (0.238) | (0.237) |
| Month dummy = March | -0.19 | -0.19 | 0.06 | 0.05 |
|  | (0.525) | (0.524) | (0.264) | (0.264) |
| Month dummy = April | -0.65 | -0.63 | -0.25 | -0.23 |
|  | (0.484) | (0.485) | (0.639) | (0.638) |
| *Rainfall* |  |  |  |  |
| Rainfall shock | 0.01 | 0.04 | 0.04 | -0.03 |
|  | (0.089) | (0.129) | (0.050) | (0.072) |
| Year=2011 | -0.08 | -0.08 | -0.04 | -0.04 |
|  | (0.470) | (0.470) | (0.238) | (0.237) |
| Constant | -0.66 | -0.67 | -0.48* | -0.48* |
|  | (0.518) | (0.519) | (0.286) | (0.286) |
|  |  |  |  |  |
| Observations | 1,218 | 1,218 | 2,659 | 2,659 |
| R-squared | 0.019 | 0.019 | 0.009 | 0.009 |
| *** p<0.01, ** p<0.05, * p<0.1 |  |  |  |  |
